# Impact of Neurofascin on Chronic Inflammatory Demyelinating Polyneuropathy *via* Changing the Node of Ranvier Function: A Review

**DOI:** 10.3389/fnmol.2021.779385

**Published:** 2021-12-16

**Authors:** Ying Gao, Lingxin Kong, Shan Liu, Kangding Liu, Jie Zhu

**Affiliations:** ^1^Neuroscience Center, Department of Neurology, The First Hospital of Jilin University, Jilin University, Changchun, China; ^2^Department of Neurobiology, Care Sciences and Society, Karolinska Institute, Karolinska University Hospital Solna, Stockholm, Sweden

**Keywords:** chronic inflammatory demyelinating polyneuropathy, blood-nerve barrier, neurofascin, antibodies, node of Ranvier, IgG4

## Abstract

The effective conduction of action potential in the peripheral nervous system depends on the structural and functional integrity of the node of Ranvier and paranode. Neurofascin (NF) plays an important role in the conduction of action potential in a saltatory manner. Two subtypes of NF, NF186, and NF155, are involved in the structure of the node of Ranvier. In patients with chronic inflammatory demyelinating polyneuropathy (CIDP), anti-NF antibodies are produced when immunomodulatory dysfunction occurs, which interferes with the conduction of action potential and is considered the main pathogenic factor of CIDP. In this study, we describe the assembling mechanism and anatomical structure of the node of Ranvier and the necessary cell adhesion molecules for its physiological function. The main points of this study are that we summarized the recent studies on the role of anti-NF antibodies in the changes in the node of Ranvier function and its impact on clinical manifestations and analyzed the possible mechanisms underlying the pathogenesis of CIDP.

## Introduction

Chronic inflammatory demyelinating polyneuropathy (CIDP) is an autoimmune-mediated chronic inflammatory demyelinating disease, which usually has 6–8 weeks course before the onset of neurological symptoms. The typical clinical manifestations of CIDP include a progressive relapsing-remitting pattern in the extremities, symmetric paresthesias and weakness, areflexia, cranial nerve involvement, autonomic symptoms, and less commonly neuropathic pain. Chemical analysis of the cerebrospinal fluid of patients reveals the cell-protein separation phenomenon and electromyography indicates demyelinating or axon damage. Atypical manifestations of CIDP include simple motor dysfunction, simple sensory dysfunction, and Lewis–Sumner syndrome (multifocal demyelinating sensory and motor neuropathy with persistent conduction block), for which conventional immune therapy is not effective (Kuwabara et al., 2015, 2019). The prevalence of CIDP varies by country because of different diagnostic criteria used; it is estimated to be 1.6–8.9 per 100,000 adults (Broers et al., 2019; Lehmann et al., 2019). The prevalence rate increases with age and men are significantly more affected than women. Most patients have a history of non-specific upper respiratory tract or gastrointestinal infection within 6 weeks of the onset of neurological symptoms, while others have had hepatitis virus, HIV infection, or vaccination (Rodríguez et al., 2019). Some individuals overreact to such infections due to immune system dysregulation, resulting in the production of autoantibodies that inappropriately recognize normal molecules in the node of Ranvier, leading to peripheral neuropathies. In the recent years, antibodies to neurofascin 155 (NF155), contactin 1 (CNTN1), contactin-associated protein 1 (CASPR1), and neurofascin 186 (NF186) have been implicated in the pathogenesis of CIDP *via* functional impairment of the node of Ranvier.

The structure of the nervous system is similar to that of a cable transmission system. With respect to the myelinated axons, the nodes of Ranvier act as repeaters to regenerate the action potential, as they propagate in a saltatory manner along the axon to the terminal nerve and significantly increase the velocity of action potential conduction (Huxley and Stampfli, 1949; Cohen et al., 2020). NF plays an important role in the assembly process and maintains the functional stability of the node of Ranvier. Previous studies have confirmed that autoantibodies are involved in the pathogenesis of CIDP including antibodies against NF, CASPR1, and CNTN1 (Ng et al., 2012; Delmont et al., 2017; Cortese et al., 2020). A dysfunction of the blood-nerve barrier (BNB) exposes the antigens of the peripheral nervous system (PNS), which activate the immune response to cluster immune cells, secrete cytokines, and produce antibodies (Mathey et al., 2015). Compared to cellular immunity, humoral immunity is more significant in the pathogenesis of CIDP by producing anti-NF antibodies.

Neurofascin comprises two subtypes such as NF186 and NF155. Due to the diverse functions and structures of each subtype of immunoglobulin (Ig) and the different anatomical features of the paranode and node, the manifestation and therapy of anti-NF155 antibody-positive CIDP are different from those of anti-NF186 antibody-positive CIDP (Ogata et al., 2015; Kira, 2021). In this study, we mainly discuss the effects of NF on the assembly and maintenance of the node of Ranvier, the role of anti-NF antibodies in the pathogenesis of CIDP, and the corresponding characteristic manifestation of the mechanism.

## Structure of the Node of Ranvier

**FIGURE 1:**
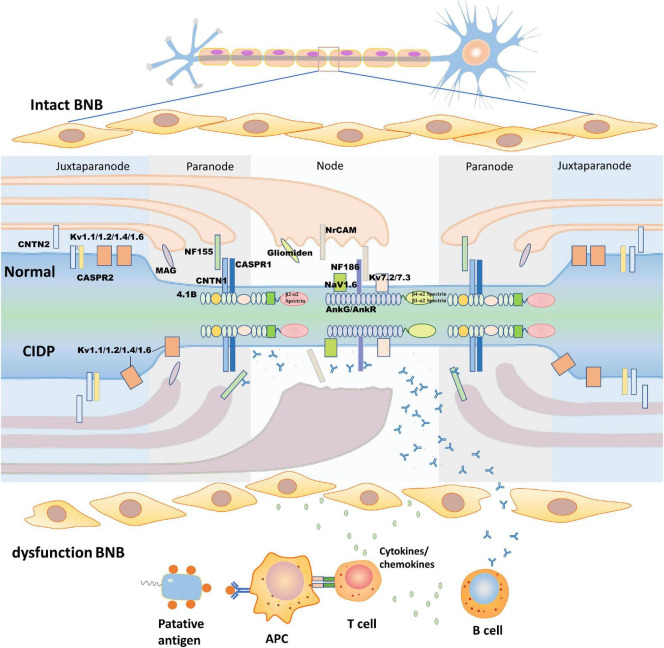
Structure of the node of Ranvier and the pathogenic process of chronic inflammatory demyelinating polyneuropathy (CIDP). The upper half of this figure shows the morphological structure of the node of Ranvier. According to their molecular composition and function, the node of Ranvier is divided into four parts: the node, paranode, juxtaparanode, and internode, which is between two juxtaparanodes and is not shown in the figure. In this study, we describe the first three. The node (mainly NF186, NrCAM, gliomedin, NaV, and Kv) and juxtaparanode (mainly Kv, CNTN2, and CASPR2) have high densities of potassium ion channels to ensure depolarization and repolarization. The paranode (mainly NF155, CNTN1, and CASPR1) acts as a septate-like junction without ion channels. The lower half of this figure shows putative pathologic changes in the node of Ranvier in CIDP. As blood-nerve barrier (BNB) dysfunction occurs, the putative antigen is processed by antigen-presenting cells to T cells, which activate B cells to produce antibodies by secreting cytokines/chemokines. The antibodies pass through the damaged BNB and then bind to the epitope of the antigen with the assistance of cytokines/chemokines. The formation of antigen-antibody complexes disrupt the structure of the node of Ranvier, concentration of ion channels, and damage Schwann cell microvilli. CASPR, contactin-associated protein; CNTN, contactin; Kv, voltage-gated potassium channel; MAG, myelin-associated glycoprotein; Nav, voltage-gated sodium channel; NF, neurofascin; NrCAM, neuronal cell adhesion molecule.

In humans, myelin is applied to most nerve fibers in the PNS by Schwann cells. To some extent, the involved nerves in CIDP are influenced by the anatomical differences in the peripheral nerves. A study of 9 patients with anti-NF155 antibody-positive CIDP showed that the median and ulnar nerves are more vulnerable than the sural sensory nerves, which are consistent with their different structures. Moreover, conduction studies on the median and ulnar nerves show that NF autoantibodies affect the properties of the nerve terminals, while those on the sural nerves show that NF autoantibodies affect the intermediate nerve segment (Kuwabara, 2007; Ogata et al., 2015). These autoantibodies often preferentially attack sites where the BNB is anatomically deficient or leaky (Olsson, 1990). The myelinated sheath is a multilamellar sheet of Schwann cell membrane that wraps around axons to increase transmembrane resistance and decrease membrane capacitance, which can be divided into four parts according to structural features: the nodes of Ranvier, paranode, juxtaparanode, and internode (Figure 1; Lambert et al., 1997; Pedraza et al., 2001; Rasband and Peles, 2015). The node of Ranvier is located in the gap between two segments of the myelin sheath, which is not completely naked and leaky, but is covered by the outermost layer of Schwann cell microvilli (Berthold et al., 1983). There are NF186, sodium ion channels (NaV), potassium ion channels (including TRAAK, TREK1, Kv7.2/Kv7.3, and Kv3.1b), and cytoskeletal protein ankyrinG (AnkG)/β4-α2 spectrin or ankyrinR (AnkR)/β1-α2 spectrin on the axon side of the node of Ranvier (Cooper, 2011; Ho et al., 2014). The main molecules in the microvilli of Schwann cells are neuronal cell adhesion molecules (NrCAMs) and gliomedin, both of which exist as secreted proteins in the gap between Schwann cells and axons (Davis et al., 1996; Eshed et al., 2005) to promote the process of NF186 concentration and node assembly (Eshed et al., 2005; Feinberg et al., 2010; Labasque et al., 2011). The paranode is a barrier structure that restricts the free movement of molecules in the two flanks and primarily comprises three molecules, NF155 on the Schwann cell and CASPR1 and CNTN1 on the axon. The paranode function depends on the integrity of the complex (Bhat et al., 2001; Boyle et al., 2001; Gollan et al., 2002; Pillai et al., 2009; Feinberg et al., 2010). In addition, 4.1b and β2-α2 spectrin in the axons and AnkB, 4.1 g, and β2-α2 spectrin in Schwann cells constitute paranode-skeleton components, which are involved in maintaining the structure and function of the node of Ranvier (Ogawa et al., 2006; Buttermore et al., 2011; Zhang et al., 2013). Myelin-associated glycoprotein (MAG) is located at the Schwann cell paranodal loops, internodes, and Schmidt–Lanterman incisures (Stathopoulos et al., 2015). MAG is considered to be the antigenic target of IgM–anti-MAG peripheral neuropathy associated with monoclonal gammopathy, but it has not been verified in animal experiments (Montag et al., 1994). The juxtaparanode is a complex comprising contactin 2 (CNTN2), contactin-associated protein 2 (CASPR2), and potassium ion channels (Poliak et al., 2003; Traka et al., 2003). Another molecule found in the juxtaparanode is disintegrin and metalloproteinase domain-containing protein 22 (ADAM22), which is a major neuronal receptor for leucine-rich glioma-inactivated4 (Lgi4)-mediated Schwann cell signaling (Ozkaynak et al., 2010). ADAM22-deficient mice develop ataxia and peripheral nerve hypomyelination (Sagane et al., 2005). Besides, the cytoskeletal protein β2-α2 spectrin and postsynaptic density protein-95/93 are unnecessary for potassium ion channel clustering in the juxtaparanode and their functions are unclear (Ogawa et al., 2010). The integrity of the juxtaparanode, which depends on the integrity of the paranode, is important for maintaining the stability of the resting potential and electrical conduction of the internode (Wang et al., 1993; Rasband, 2010). The paranode has been viewed as a “fence” of membrane protein that restricts the diffusion of potassium ion channels into the juxtaparanode. When any molecular species belonging to the “fence” is absent, the potassium ion channels spread to the juxtaparanode (Poliak and Peles, 2003; Salzer, 2003).

## Assembly of the Node of Ranvier

All the types of glial cells found in the PNS originate from neural crest cells, with gliogenesis starting at embryonic day 11 in the mouse (Jacob, 2015). Two types of glia are generated from neural crest cells such as satellite glia and Schwann cell precursors. The latter differentiate into Schwann cells or non-myelinating Schwann cells, such as melanocytes, parasympathetic neurons, or mesenchymal stem cells, under the control of the regulatory factors Notch/Delta, fibroblast growth factors (FGFs), and bone morphogenetic protein (BMP) (Jacob, 2015). The precise starting time of gliogenesis in the human embryo is unclear and filling this gap would be an interesting and attractive domain of study. As the differentiation of Schwann cell precursors into Schwann cells completes, the assembly of the node of Ranvier begins. The conduction of action potentials relies on rapid depolarization and repolarization, which require the differential distribution of sodium and potassium ion channels in axons. The structural basis of saltatory conduction is the integrity of the node of Ranvier and paranodal structures (Poliak and Peles, 2003; Salzer, 2003). Before the formation of the node, NF186 is evenly distributed on the axons. NF186 concentrates on node through glial-axon interaction along with two hemi-nodes fused into a node (Lambert et al., 1997; Salzer, 2003). The assembly of the node of Ranvier begins with contact between the Schwann cells and the axon. Cytokines secreted by Schwann cells, such as NrCAM and gliomedin, interact with NF186 and other adhesion molecules on the axon surface to promote the assembly of the node of Ranvier (Zhang et al., 2012). Schwann cells wrap around axons to form a myelin sheath during the myelin sheath spreading process and secrete the adhesion molecule NrCAM, which binds with gliomedin to enhance the movement of NF186 toward the hemi-node to form a new node (Amor et al., 2017). NrCAM and NF186 come from two similar Ig families and have highly similar sequences and domains (Volkmer et al., 1992); when NrCAM is absent, NaV can cluster at the node, but will be delayed considerably (Sakurai et al., 2001; Custer et al., 2003). When both the NrCAM and gliomedin are absent, the density of NaV at the node is decreased (Amor et al., 2017). The extracellular domain of NF186 contacts Schwann cell microvilli and moves toward another hemi-node through the mediation of NrCAM and gliomedin. The intracellular domain binds to AnkG, which then connects to the β4-α2 spectrin, NaV, and other cytoskeletal proteins to form a complex and continues to extend until the two hemi-nodes fuse into a node (Rasband et al., 1999; Susuki et al., 2013). Recent evidence has suggested that in addition to interacting with AnkG, NF186 can also directly interact with sodium-channel subunits (Lacas-Gervais et al., 2004). When the binding site of AnkG with NF186 is mutated or absent, the intracellular domain of NF186 cannot bind to it, which decreases the stability of NaV in the node and nerve conduction velocity (Susuki et al., 2013). AnkG deficiency is followed by a lack of β4 spectrum. At this time, AnkR and β1 spectrin will replace their roles, but they have a low affinity for cell adhesion molecules (Ho et al., 2014). In addition to participating in the assembly of the node of Ranvier, NrCAM and gliomedin are related to the axonal action potential conduction velocity in the mature node of Ranvier (Feinberg et al., 2010).

The paranode begins to assemble after the node cluster NaV and then the potassium ion channel concentrates on the juxtaparanode (Vabnick et al., 1996; Schafer et al., 2006). In the absence of NF186, NF155 can facilitate the recruitment of NaV (Zonta et al., 2008; Feinberg et al., 2010; Amor et al., 2017). CASPR1, CNTN1, and NF155 in the paranode act as transverse bands to restrict the molecules on both the sides (Sherman et al., 2005). Once one of the three molecules is dysfunctional, the barrier effect is impaired, potassium ion channels in the juxtaparanode diffuse into the paranode, and the normal saltatory conduction process is destroyed (Poliak and Peles, 2003; Salzer, 2003). NF155 on Schwann cells contacts the CNTN1-CASPR1 complex, which is located on the axon, through the extracellular molecule domain. The CNTN1-CASPR1 complex connects with the β2-α2 spectrin and actin through 4.1B to form a transverse band to complete the barrier function of the paranode and restrict the voluntary movement of other protein molecules (Rosenbluth, 2009; Horresh et al., 2010; Ogawa et al., 2010). In summary, the roles of the paranode are as follows: (1) to construct a septate-like transverse band by cell adhesion molecules and cytoskeleton proteins to restrict the voluntary movement of molecules on both the sides of the paranode and increase the electrical resistance between the internode and node, which is the basic structure of saltatory conduction; (2) to promote and maintain the stability of the node. The paranode can compensate for the assembly of the node and cluster NaV through NF155 when NF186 is absent; and (3) to connect axons and myelin sheaths to promote the process of the early node of Ranvier assembly.

## Role of Neurofascin in the Assembly and Maintenance of the Node of Ranvier

### Isoform of Neurofascin

Neurofascin, a neural-cell surface protein, is part of the L1 group of the immunoglobulin superfamily, including L1, close homolog of L1 (CHL1), and NrCAM (Liu et al., 2011). Different polypeptides of 155, 166, 180, and 186 kDa are produced by alternative splicing in the isoform of NF (Kriebel et al., 2012). All are composed of six Ig-like domains, three to four fibronectin type III (FNIII), the proline-, alanine-, threonine (PAT)-rich domain (also referred to as mucin-like domain), and a transmembrane domain (Hassel et al., 1997). The specific function of each domain in different isoforms of NFs is not clear so far and further investigation is necessary in the future. NF180 and NF166 are expressed in the surface of immature neurons. NF166 is composed of six Ig-like domains, FNIII domains 1, 2, and 4 without the PAT domain, and is expressed in the developing chick dorsal root ganglia. NF180 is different from NF166 in terms of its addition to the composition of the PAT domain. Evidence has shown that the NF180 isoform is expressed in the embryonic brain and during early development in the rat brain (Hassel et al., 1997; Burkarth et al., 2007). However, the specific functions of NF180 and NF 166 remain unclear and additional investigation is needed. The difference between NF155 and NF186 relies on the extracellular domains. NF155 carries fibronectin type 3 (FN3); however, NF186 lacks this domain and instead has FN5 and the PAT domain between FN4 and FN5. NF is indispensable for the intact structure and function of axons and the structures of nodes and paranodes in mice whose NF gene is knocked out and cannot be assembled properly. NaV is diffusely distributed in axons, the septate-like transverse band effect of paranode disrupts, and nerve impulses cannot be transmitted in a saltatory manner (Sherman et al., 2005). NF is also expressed in human kidney glomeruli besides the nervous system (Sistani et al., 2013), which was also verified by a study of two patients with anti-NF186 antibody-positive CIDP who presented with nephrotic syndrome in the meantime (Delmont et al., 2017).

The PAT domain is thought to confer an extended and more flexible structure of NF186, which can enhance the interactions of NF186 with gliomedin and NrCAM and enable NF186 to target at the node and guide NaV and AnkG located at proper locations. When NF186 is absent, the nodal gap shortens progressively and AnkG and Nav disappear from the axon, resulting in the deduction of conduction velocity. Moreover, NF186 coordinates nodal organization and the enrichment of both the neuron-specific proteins and glial-specific proteins to nodes in PNS myelinated axons and acts as a barrier to restrict the invasion of flanking paranodal domains in myelinated axons (Thaxton et al., 2011).

### Function of Neurofascin 155 in the Node of Ranvier

Paranode stability requires interactions between glial NF155 and the CNTN1-CASPR1 complex, which is expressed on the axonal membrane (Charles et al., 2002). There are three primary molecules involved in the paranode: NF155, CASPR1, and CNTN1. NF155 is essential for maintaining ion channel-related proteins at the proper position in the axons. NF155 plays an important role in the paranode-assembly mechanism by clustering CASPR1 and CNTN1 through the extracellular domain. When NF155 is malfunctioning, the total quantity of CASPR1 and CNTN1 does not decrease, but they cannot concentrate on the paranode, leading to paranode structure damage and NaV channels disorderly diffuse on the axon. The order of saltatory conduction is disrupted and conduction velocity decreases (Sherman et al., 2005). NF155 is not indispensable for node assembly, but it is important for the stability of the node of Ranvier. Once NF155 is attacked, the restrictive effect of the paranode is disrupted and the potassium ion channel in the juxtaparanode invades the node, resulting in the disruption of the proper ion channel distribution and saltatory conduction (Zonta et al., 2008). Additionally, NF155 has a vital function in maintaining the stability of nodes; compared with the loss of NF186 alone and when both the NF186 and NF155 are lost at the same time, the action potential conduction velocity and the stability of the node decrease and the axon degeneration is intensified (Taylor et al., 2017).

In summary, NF is indispensable for the structural integrity of the node of Ranvier and paranode, which is the basis for saltatory conduction of action potentials. Both the NF186 and NF155 play separate roles in the myelin sheath: NF155 is a component of the paranode that stabilizes ion channels located in two flanks of the paranode, whereas NF186 acts as a barrier to restrict the invasion of flanking paranodal domains in myelinated axons.

## Role of the Node of Ranvier and Neurofascin in Chronic Inflammatory Demyelinating Polyneuropathy

### Isoforms of Neurofascin as the Immune Targets in Chronic Inflammatory Demyelinating Polyneuropathy

Some patients with CIDP have infections after the onset of neurological symptoms, but so far, no causative pathogens have been found to be related to the occurrence of the disease. CIDP is considered an autoimmune disease and may be treated and improved by effective targeting of the autoimmune response and the therapeutic methods include intravenous immunoglobulin (IVIg), plasma exchange, and corticosteroids, which have been proven to inhibit the inflammatory response in the blood circulatory system and peripheral nerves (Querol et al., 2017). There are many target antigens in CIDP including NF, CASPR1, CNTN1, and gliomedin. Both the cellular and humoral immunity are involved in the pathogenesis of CIDP. At onset, cytokines secreted from T cells trigger inflammation of the BNB, which accelerates the exposure of autoantigens such as NF186 and NF155 to circulating immune cells and activates humoral immunity (Ubogu, 2015). The NF antibodies play a crucial role in the pathogenesis because of the characteristic position and function of NF in axons and glial cells (Rasband and Peles, 2021). With respect to the humoral immune response, the immunoglobulin G4 (IgG4) subclass was predominant in antibodies to NF in CIDP and has characteristic clinical manifestations, while other subclasses of IgG also participate in this process (Ogata et al., 2015; Burnor et al., 2018).

### Features of Antibodies in Anti-neurofascin Antibody-Positive Chronic Inflammatory Demyelinating Polyneuropathy

The isoform of NF155 is more vulnerable to attack in immune modulatory dysfunction than other molecules in CIDP (Delmont et al., 2017; Burnor et al., 2018; Kira, 2021). The IgG subclass was more frequently detected in both the anti-NF186 and anti-NF155 antibody-positive CIDP. The IgG4 subtype is predominant in patients with CIDP with anti-NF155 antibody positivity, but IgG3 and IgG1 take advantage of IgG4 in patients with CIDP with anti-NF186 antibody positivity (Ng et al., 2012; Querol et al., 2017; Kira et al., 2019). Ig is progressively produced by maturing B cells in a sequential order (IgM→IgG3→IgG1→IgG2→IgG4) (Collins and Jackson, 2013), which is in accordance with the fact that both the positive rate and titer of IgG4 are predominant in CIDP, but IgM and IgG3 are more detectable in Guillain-Barré Syndrome (GBS) (Burnor et al., 2018). NF186 is more accessible to be attacked by antibodies in circulation than NF155 because of its anatomical features (Lonigro and Devaux, 2009). Anti-NF antibodies are found in about 4–18% in patients with CIDP and acute inflammatory demyelinative polyradiculoneuropathy (AIDP) (Ng et al., 2012; Ogata et al., 2015); the positive rate of NF186 is lower than that of NF155 in CIDP, which is probably due to the paranode maintenance effect to sodium channels. Due to the unique feature of IgG4, the positive rate of anti-NF155 antibodies is approximately 25% in IVIg-resistant patients with CIDP. The paranode restricts NaV in nodes when NF186 is decreased or absent (Lonigro and Devaux, 2009).

**FIGURE 2:**
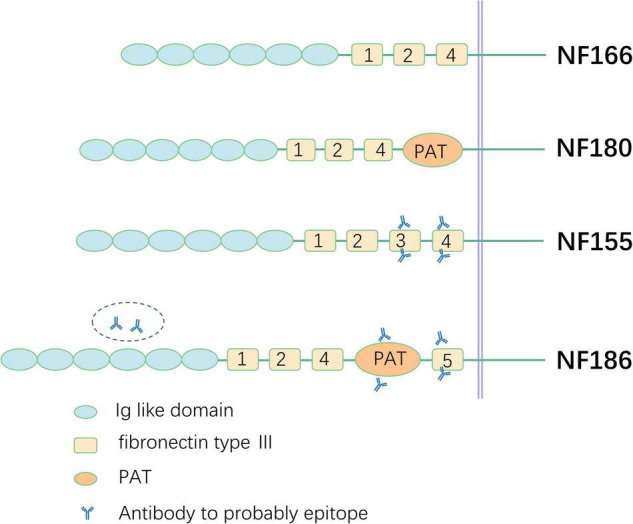
Schematic illustration of different neurofascin isoforms and epitope. PAT, proline-, alanine-, threonine-rich.

FN3 is considered an antigenic determinant of NF155 in CIDP and other inflammatory demyelinating polyneuropathies (Delmont et al., 2017; Burnor et al., 2018). However, in another study, FN3 to FN4 domains were confirmed as targets for NF155-specific reactivity (Ng et al., 2012). Both the FN5 (Ng et al., 2012) domain and Ig domain (Delmont et al., 2017) are considered the target epitopes of NF186 (Figure 2), which require further study in the future.

### Dysfunction of the Blood-Nerve Barrier Is the Early Feature in Pathology of Chronic Inflammatory Demyelinating Polyneuropathy

The BNB directly communicates with the circulating blood and nerve lumen and consists of simple endoneurial microvascular endothelium cells, which are connected by intercellular tight junction. It is a selectively permeable barrier that increases transendothelial electrical resistance, regulates the diffusion of molecules and nutrients, and restricts hematogenous cells from invading nerves (Ubogu, 2020). The endothelial cells share the basement membrane with the surrounding cells called pericytes, which are considered to play a significant part in peripheral neuropathy. An intact BNB is required for effective nerve conduction to provide a normal endoneurial homeostasis. In case of infection, trauma, or dysfunction of the immune system, cytokines and immune cells disrupt the cellular tight junctions and change the permeability of the BNB. Ultrastructural examination of endoneurial microvascular endothelium cells in patients with GBS and CIDP revealed the presence of tight junctions between leukocytes and endothelial cells, which change permeability of the BNB and disrupt endoneurial homeostasis (Bosetti et al., 2016; Dong et al., 2016).

The BNB dysfunction is the first step in the pathological cascade of CIDP. Both the cellular and humoral immunities are involved in the pathogenesis of CIDP. Previous studies have demonstrated that the filtration of autoreactive T-cells, macrophages, and cytokines leads to dysfunction of the BNB, which makes it accessible to antibodies in circulation (Figure 1; Lonigro and Devaux, 2009). A study showed that injection of anti-NF antibodies from patients with CIDP to the experimental autoimmune neuritis (EAN) model could enhance and prolong an ongoing neuritis, but injection of anti-NF antibodies to the control group is not pathogenic (Ng et al., 2012). An electrophysiology test of CIDP showed that distal and F-wave latencies are influenced more severely than the motor conduction velocities and compound muscle action potential amplitudes and have a high frequency of spinal root hypertrophy on MRI images, which suggest that nerve terminals, major plexuses, and spinal roots are more frequently involved in patients with anti-NF155 antibody-positive CIDP, where the BNB is anatomically absent or loose (Ogata et al., 2015; Kira, 2021). The BNB maintains nerve homeostasis by preventing the free movement of soluble proteins in the circulation into the endoneurium microenvironment under normal physiological conditions. In the condition of dysfunction of the BNB (e.g., the BNB is congenitally absent or damaged by *in situ*/systematic inflammation), molecules involved in Schwann cell-axon interaction, such as NF, CNTN1, and CASPR1, are accessible to the immune system and act as putative antigens, which are presented by antigen-presenting cells through the expression of the costimulatory molecules CD80 and CD86 to T cells; in effect, T cells activate and release cytokines, including interleukin-4 (IL-4) and IL-6 (Kiefer et al., 2000; Murata and Dalakas, 2000; Hu et al., 2007). These inflammatory mediators, which increase in both the cerebrospinal fluid and serum in patients with CIDP, not only activate B cells to produce autoantibodies, but also increase the permeability of the BNB, which progressively deteriorates the local inflammatory microenvironment and assists T cells to pass through the BNB easily, resulting in further severe damage to the BNB in a vicious cycle (Kieseier et al., 2002; Chi et al., 2008). The infiltrating inflammatory cells were identified by sural nerve biopsies in CIDP including macrophages, CD8+ T cells, and CD4+ T cells (Maimone et al., 1993; Schmidt et al., 1996; Mahad et al., 2002; Schneider-Hohendorf et al., 2012). Resident and recruited macrophages, activated by cytokines produced from T cells, invade the myelin fiber *via* their Fc receptor, causing macrophage-mediated demyelination, which are the predominant infiltrating inflammatory cells in patients with CIDP. With dysfunction of the BNB, cytokines/chemokines induce cell infiltration into the nerve microenvironment and make antibodies accessible to the antigen targets of axons or Schwann cells. However, previous studies revealed that biopsied sural nerves from two anti-NF155 antibody-positive patients with CIDP demonstrated subperineurial edema and occasional paranodal demyelination, but no vasculitis, inflammatory cell infiltrates, or onion bulbs (Ogata et al., 2015; Koike et al., 2017). Another study showed that large myelinated fiber loss without cellular infiltration was observed in anti-NF186 antibody-positive patients with CIDP (Pascual-Goñi et al., 2019), which probably indicates that humoral immunity, not cellular immunity, is the primary mechanism in anti-NF antibody-positive CIDP.

### Characteristics of Anti-neurofascin Antibody-Positive Chronic Inflammatory Demyelinating Polyneuropathy

When the pathological changes of CIDP damage the paranode, severe clinical manifestations are observed with anti-NF186 antibody-positive CIDP, which is not as severe as anti-NF155 antibody-positive CIDP. Some studies have indicated that anti-NF155 antibodies are pathogenic through block NF155 and CNTN1-CASPR1 complex interaction, which is verified by the sural nerve biopsy presenting Schwann cell terminal loop detachment from axons without inflammatory infiltration (Ogata et al., 2015). Electron microscopy of anti-NF155 antibody-positive CIDP also revealed detachment of terminal Schwann cell loops from axons at the paranodes, which resulted in the disruption of septate-like transverse bands (Koike et al., 2017; Kuwahara et al., 2018). In patient with CIPD with anti-NF186 antibodies, all the microvilli of the checked nodes of Ranvier completely disappeared and the outermost cytoplasm parts of two adjacent Schwann cells tended to spread along the nodal axolemma, leading to the complete block of the nodal gap. Electron microscopy showed the disappearance of microvilli that were replaced by elongated extensions of Schwann cell cytoplasm occluding the node of Ranvier, which disturbs the NaV position and leads to the failure of impulse conduction (Vallat et al., 2018). The passive transfer of anti-NF antibodies into mice with EAN strongly exacerbates the severity of the pathology (Yan et al., 2014).

Immunoglobulin G4 exists in a monovalent bispecific form through a process termed Fab-arm exchange (Huijbers et al., 2015), which results in the inability to internalize the target antigen (Aalberse and Schuurman, 2002). IgG4 is the least abundant in serum at approximately 5% of the total four IgG subclasses and accounts for the majority of anti-NF155 antibody-positive CIDP cases (Delmont et al., 2017; Kira et al., 2019). IgG4 cannot activate complement with a compact structure, which results in inaccessibility for binding with C1q. Complements have no access to the combination of IgG4 because of the trans heavy chain CH1–CH2 domain interaction of IgG4 (Huijbers et al., 2015; Kira et al., 2019). IgG4 is produced by chronic or long-term stimulation by antigens in a non-infectious initiation and then may become the dominant subtype; IgG4 alleviates allergic inflammation by blocking the binding site of allergen-specific IgE to allergens (Huijbers et al., 2015), which suggests that IgG4 causes pathological changes by blocking protein–protein interactions. Two biopsied sural nerve specimens from patients with anti-NF155 antibody-positive IgG4-predominant CIDP showed occasional paranodal demyelination and subperineural edema, but no inflammatory cell infiltrates, onion bulbs, or vasculitis (Ogata et al., 2015). The pathogenic mechanism of anti-NF155 antibodies blocks the interaction between NF155 and the CNTN1-CASPR1 complex, resulting in saltatory conduction failure but without inducing inflammation (Kira, 2021). An electron microscopy study showed the disappearance of microvilli in the sural nerve biopsy specimen from an anti-NF186 IgG3 antibody patient with CIDP, which were replaced by elongated extensions of Schwann cell cytoplasm, so that the nodal gap was occluded (Vallat et al., 2018). IgG4 autoantibody-mediated disease has strong association with human leukocyte antigen (HLA) class II alleles. A recent report showed that the frequency of the HLA-DRB1*15 allele was significantly higher in 13 patients with NF155 + CIDP from European countries (Spain, France, Italy, and the United Kingdom; 92% Caucasians) than in the control Spanish populations (Martinez-Martinez et al., 2017). However, a Japanese study showed that all the 22 patients with IgG4 anti-NF155 antibody-positive CIDP had clearly high frequencies of HLA-DRB1*15, -DRB1*15:01, -DQB1*06:01/06:02, -DQB1*06:02, and -DRB1*15:01-DQB1*06:02 (Ogata et al., 2020).

### Clinical Manifestations of Anti-neurofascin Antibody-Positive Chronic Inflammatory Demyelinating Polyneuropathy

Patients with immunoglobulin G4-predominant anti-NF155 antibody-positive CIDP are often refractory to treatment with IVIg, but they partially respond to rituximab and corticosteroid treatment because IgG4 does not fix complements or bind to Ig receptors in a monovalent bispecific form *in vivo* (Burnor et al., 2018; Pascual-Goñi et al., 2019). Most anti-NF186 antibody-positive CIDPs are responsive to IVIg, probably because IgG4 is not the predominant subtype and the location of NF186 is more accessible to Ig in circulation (Lonigro and Devaux, 2009; Delmont et al., 2017). Another common feature of IgG4-mediated diseases is their positive response to B-cell depletion treatment (Querol et al., 2017). IgG4 antibodies are produced by regulatory B (Breg) cells (van de Veen et al., 2013). The inhibitory Ig receptor low-affinity IgG Fc region receptor IIb (FcγRIIB) is a major mediator of the IVIg response. Gene expression profiling suggests that IL-10-positive Breg cells have reduced expression of FcγRIIB compared to IL-10-negative Breg cells (Lünemann et al., 2015). This difference could partly explain the IVIg resistance, but B cell depletion was efficient.

The clinical features of anti-NF155 antibody-positive CIDP include younger age at onset, predominant distal limb weakness, high-amplitude and low-frequency tremors, ataxia with cerebellar features, and a higher prevalence of poor response to IVIG when compared with seronegative patients (Querol et al., 2014; Kadoya et al., 2016). Although tremor and ataxia accompanied by cerebellar features commonly occurred in anti-NF155 antibody-positive CIDP, there is no evidence of abnormalities on MRI of the head in anti-NF155 antibody-positive CIDP. Several studies have reported that MRI scans of the cervical and lumbosacral nerves show enlarged nerve roots and proximal nerve segments (Kira, 2021). The clinical features of anti-NF186 antibody-positive CIDP are different from those of anti-NF155 antibody-positive CIDP, which include subacute onset, sensory ataxia, conduction block, and cranial nerve involvement. In comparison with anti-NF155 antibody-positive CIDP, most patients with anti-NF186 antibody positivity showed a good response to IVIg and corticosteroid treatment. None of the patients showed tremor or neuropathic pain (Delmont et al., 2017).

## Conclusion

Recently, an increasing number of studies have revealed that the assembly and maintenance of the node of Ranvier depends on the normal functions of various molecules on the node, paranode, and juxtaparanode including NrCAM, gliomedin, CNTN1/2, CASPR1/2, MAG, and NF. These molecules keep the ion channels in proper positions to ensure that the action potential is conducted in a saltatory manner. The BNB dysfunction is considered the initiation of pathology of CIDP and passive transfer of anti-NF antibodies to EAN could aggravate and delay ongoing neuritis. Antigenic targets should be accessible to antibodies, which are realized by cytokines and immune cell infiltration, resulting in opening the BNB and providing access to autoantibodies. The immune response is activated by the exposure of autoantigens or foreign antigens, resulting in the production of cytokines and antibodies to resist the invasion of the “foreigner.” These immune mediators could disrupt the physiological effects of the node of Ranvier and lead to the occurrence of diseases such as CIDP, AIDP, and combined central and peripheral demyelination. Antibodies to both the NF155 and NF186 are involved in the pathogenesis of CIDP, but Ig subclasses and clinical manifestations are significantly different. The positive rate of the anti-NF155 antibody was higher than that of NF186 in CIDP probably because NF155 can partly compensate for the function of NF186. The mechanism by which autoantibodies belonging to the same IgG4 subclass can cause IgG4 antibody-specific disease features and different responses to conventional immunotherapy requires further study and it could guide the development of more efficient treatments and avoid unnecessary therapy. The sequential order of Ig indicates that the appearance of IgG4 results from long “foreign” stimulation and the production of IgG3 and IgM is an acute immune response. Unique diagnosis and treatment strategies are required for IgG4-related neuropathy. Previous studies indicate that IgG4-related CIDP is poorly responsive to IVIg, but no multicenter studies have focused on the effect of immunosuppressors in patients with CIDP. Further multicenter studies are necessary to clarify the clinical characteristics of the autoantibody subtypes, which assist diagnosis and the choice of therapeutic strategies. Further, studies are needed to reveal the mechanism of the different responses of anti-NF186/-NF155 IgG4-positive CIDP to IVIg. Although we have discussed in depth the mechanism underlying the pathogenesis of NF in CIDP, many problems remain unsolved in this field. Further study will be essential for understanding the specific function of each domain in the different isoforms of NF, how the NFs interact with their corresponding receptors/ligands, the factors that trigger dysfunction of the BNB, the role of the pericytes in pathology of CIDP, and the different modifications of the BNB that occur in the various subtypes of CIDP.

## Author Contributions

YG conceived and prepared the manuscript. LK, SL, and KL prepared the manuscript and the figures. JZ helped to conceive and reviewed the manuscript. All the authors read and approved the final manuscript.

## Conflict of Interest

The authors declare that the research was conducted in the absence of any commercial or financial relationships that could be construed as a potential conflict of interest.

## Publisher’s Note

All claims expressed in this article are solely those of the authors and do not necessarily represent those of their affiliated organizations, or those of the publisher, the editors and the reviewers. Any product that may be evaluated in this article, or claim that may be made by its manufacturer, is not guaranteed or endorsed by the publisher.
