# Supplementary material for: Impact of Neurofascin on Chronic Inflammatory Demyelinating Polyneuropathy via Changing the Node of Ranvier Function: A Review
Source: Front Mol Neurosci. 2021 Dec 16;14:779385. doi: 10.3389/fnmol.2021.779385 (PMC8716720; doi:10.3389/fnmol.2021.779385)
Supplement: Supplementary file 1 [file Image_1.pdf]

Supplementary Material

Supplementary Figures

Fig1:

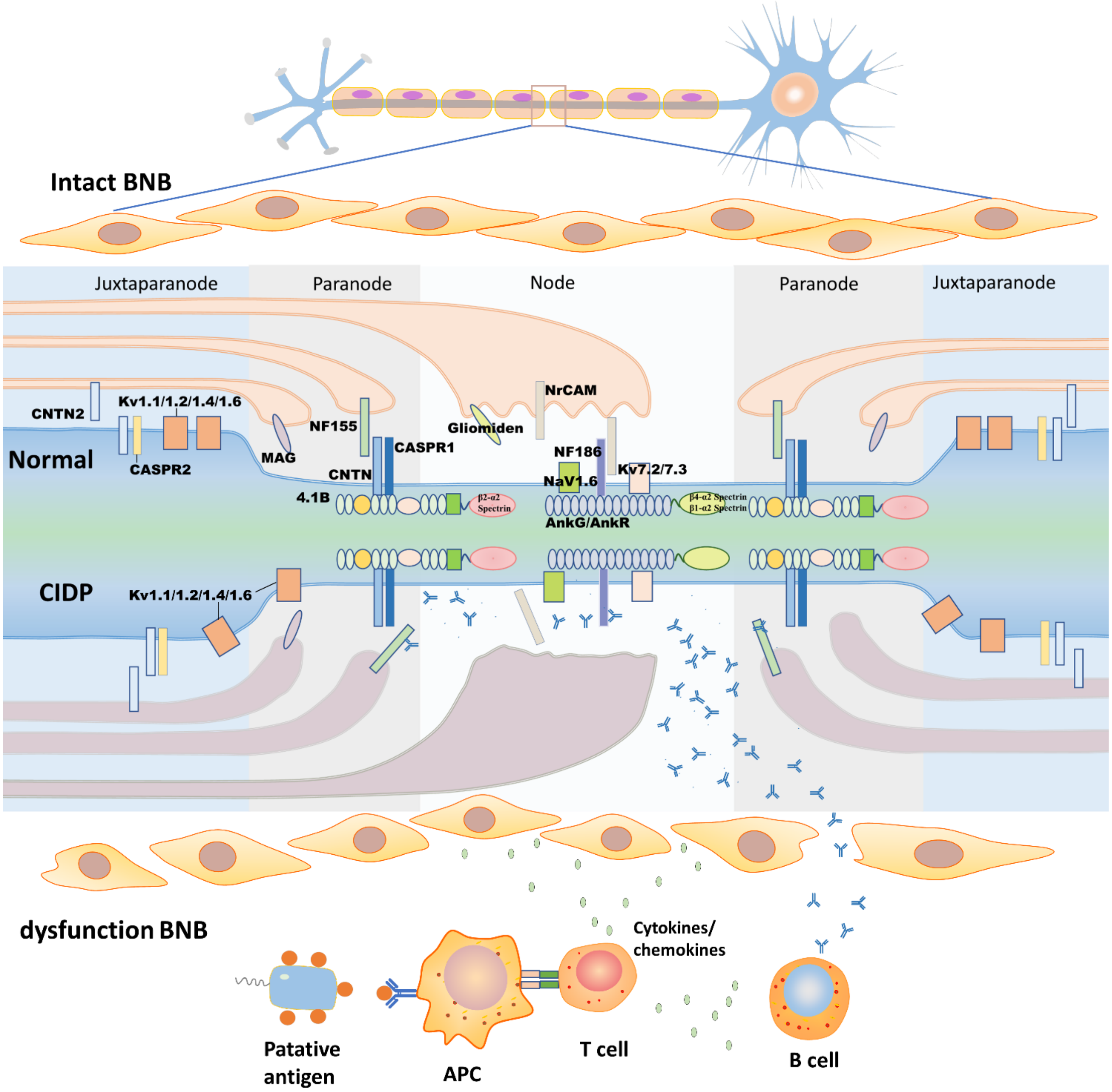

Fig1: the structure of node of Ranvier and pathogenic process of CIDP

Fig1: Structure of the node of Ranvier and the pathogenic process of chronic inflammatory demyelinating polyneuropathy (CIDP)

The upper half of this figure shows the morphological structure of the node of Ranvier. According to their molecular composition and function, the node of Ranvier is divided into four parts: the node, paranode, juxtaparanode, and internode, which is between two juxtaparanodes and is not shown in the figure. In this study, we describe the first three. The node (mainly NF186, NrCAM, gliomedin, NaV, and Kv) and juxtaparanode (mainly Kv, CNTN2, and CASPR2) have high densities of potassium ion channels to ensure depolarization and repolarization. The paranode (mainly NF155, CNTN1, and CASPR1) acts as a septate-like junction without ion channels.

The lower half of this figure shows putative pathologic changes in the node of Ranvier in CIDP. As blood-brain barrier (BNB) dysfunction occurs, the putative antigen is processed by antigen-presenting cells to T cells, which activate B cells to produce antibodies by secreting cytokines/chemokines. The antibodies pass through the damaged BNB and then bind to the epitope of the antigen with the assistance of cytokines/chemokines. The formation of antigen-antibody complexes disrupt the structure of the node of Ranvier, concentration of ion channels, and damage Schwann cell microvilli.

Abbreviations: CASPR, contactin-associated protein; CNTN, contactin; Kv, voltage-gated potassium channel; MAG, myelin-associated glycoprotein; Nav, voltage-gated sodium channel; NF, neurofascin; NrCAM, neuronal cell adhesion molecule

Fig2

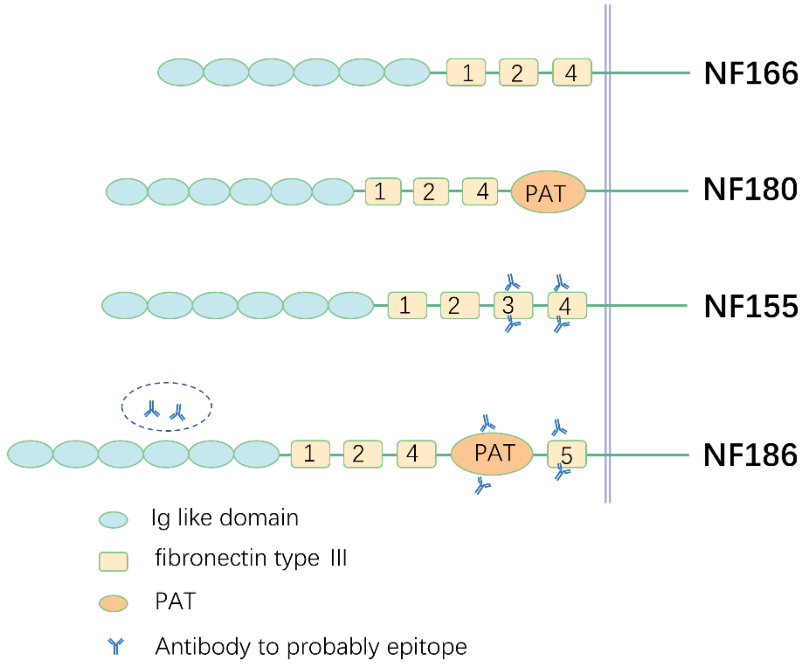

Fig2: Schematic illustration of different neurofascin isoforms and epitope

PAT, proline-, alanine-, threonine-rich
